# Exceptional dinosaur fossils reveal early origin of avian-style digestion

**DOI:** 10.1038/s41598-018-32202-x

**Published:** 2018-09-21

**Authors:** Xiaoting Zheng, Xiaoli Wang, Corwin Sullivan, Xiaomei Zhang, Fucheng Zhang, Yan Wang, Feng Li, Xing Xu

**Affiliations:** 1 Institute of Geology and Paleontology, Linyi University, Linyi City, Shandong 276005 China; 2 Shandong Tianyu Museum of Nature, Pingyi, Shandong 273300 China; 3 Department of Biological Sciences, University of Alberta, Edmonton, Alberta T6G 2E9 Canada; 4 Philip J. Currie Dinosaur Museum, Wembley, Alberta T0H 3S0 Canada; 5 University of Chinese Academy of Sciences, Beijing, 100049 People’s Republic of China; 6 Key Laboratory of Vertebrate Evolution and Human Origins of Chinese Academy of Sciences, Institute of Vertebrate Paleontology and Paleoanthropology, Chinese Academy of Sciences, Beijing, 100044 China

## Abstract

Birds have a highly specialized and efficient digestive system, but when this system originated remains uncertain. Here we report six gastric pellets attributable to the recently discovered 160-million-year-old troodontid dinosaur *Anchiornis*, which is among the key taxa for understanding the transition to birds. The gastric pellets contain lightly acid-etched lizard bones or fish scales, and some are associated with *Anchiornis* skeletons or even situated within the oesophagus. *Anchiornis* is the earliest and most basal theropod known to have produced gastric pellets. In combination with other lines of evidence, the pellets suggest that a digestive system resembling that of modern birds was already present in basal members of the Paraves, a clade including troodontids, dromaeosaurids, and birds, and that the evolution of modern avian digestion may have been related to the appearance of aerial locomotion in this lineage.

## Introduction

Modern birds have a highly specialized and efficient digestive system, which facilitates their high metabolism and underpins their capacity for aerial locomotion^1,2^. Several unique features characterizing this system have been recently discovered in stem birds^3–6^. For example, a few individual Early Cretaceous stem bird fossils preserve recognizable gastric pellets, representing regurgitated masses of indigestible material such as bones and feathers. In modern birds, gastric pellets form by compaction in a muscular gizzard, and are subsequently regurgitated through the oesophagus before finally being expelled from the mouth. Accordingly, their occurrence in stem birds suggests the presence of efficient antiperistalsis and a muscular gizzard in taxa of this grade^3^. Conversely, reports of bone fragments in coprolites attributable to *Tyrannosaurus* and other large theropods^7–9^ indicate that efficient antiperistalsis and other features integral to regurgitation of pellets had not yet evolved in basal coelurosaurian theropods. However, such theropods probably already possessed a two-chambered stomach incorporating a muscular gizzard as in modern birds, based on other lines of evidence including the presence of gastroliths in a wide range of non-avialan theropods^10^.

Here we report six specimens (STM0-38, STM0-179, STM0-224, STMA0-4, STM0-227, and STM0-228, all housed in the Shandong Tianyu Museum of Nature) that either include or represent apparent gastric pellets. All these specimens are from exposures of the Oxfordian Tiaojishan Formation at the Daxishan Locality, Linglongta Township, Jianchang County, Liaoning Province, China^11^.

## Results

STM0-38, STM0-179, STM0-224 and STMA0-4 are skeletal specimens assignable to the recently discovered basal paravian *Anchiornis*, which is among the key taxa for understanding the transition to birds^12–15^, based on the following combination of features: quadrate strongly inclined anteroventrally, elongate foramina in posteriorly widening groove along mandibular lateral surface, procumbent anterior dentary teeth, sternum and uncinate processes absent or not ossified, pubis moderately retroverted and with anterior margin slightly convex in lateral view, ischium extremely proximodistally short and dorsoventrally wide, large rectangular ischial obturator process separated by notch from ischial shaft, and tibiotarsus extremely long in proportional terms^12,13,16^.

**Figure 1:**
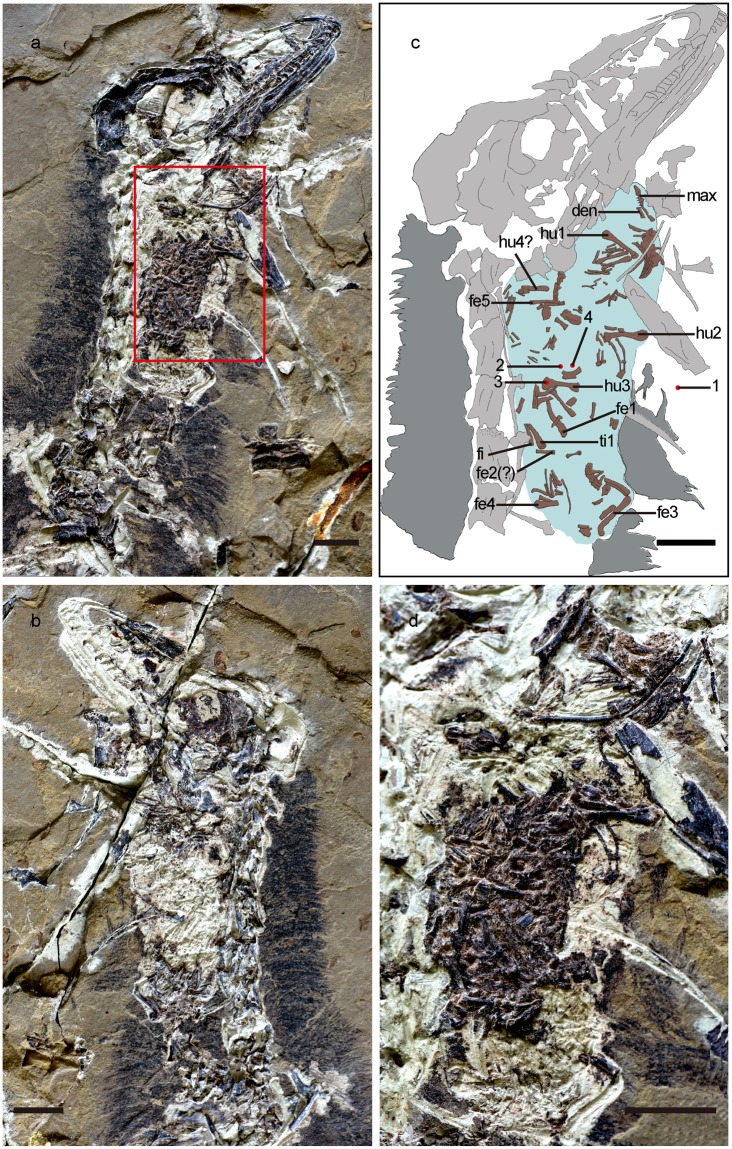
The troodontid *Anchiornis* STM0-179, with a gastric pellet comprising lizard bones preserved in the oesophageal area. (**a**) Photograph of the cranial and cervical region of the STM0-179 slab. (**b**) Photograph of the same portion of the counter slab. (**c**) Line-drawing of the cranial and cervical region of the STM0-179 slab, with the preserved pellet shaded in light blue. (**d**) Close-up photograph of the lizard bones preserved in STM0-179. In the pellet, at least three or four humeri and four or five femora are identifiable, and one of the femora is significantly smaller than the others. This suggests that the pellet contains bones from at least two relatively large lizards and one small lizard. Abbreviations: 1–4, sampling locations for the EDS analyses; den, dentary; fe1–5, femora 1–5; hu1–4, humeri 1–4; max, maxilla; ti, tibia; fi, fibula. Scale bar, 10 mm.

STM0-179 preserves a coherent oval structure, lying ventral to the anterior cervical vertebrae, that we interpret as a pellet lodged in the oesophagus (Fig. 1). The long axis of the pellet measures 70 mm, whereas the short axis measures 2.7 cm. The pellet comprises numerous lizard bones, surrounded and cemented by fine-grained white sediment probably derived from digestive residues.

The position of the pellet indicates that this structure is neither a coprolite nor simply a mass of gastric or intestinal contents. The aggregated lizard bones might conceivably represent food the dinosaur had swallowed just before its death, but several lines of evidence weigh strongly against this interpretation and in favour of the pellet interpretation. First, the aggregation contains bones from at least three individual lizards. A predatory theropod’s stomach contents might include parts of three prey animals^17–19^, but the theropod would be unlikely to have swallowed three lizards simultaneously. Second, the lizard skeletons in this aggregation are partly disarticulated. Judging by evidence from carnivorous extant birds^20^, a pellet might contain a mixture of disarticulated and/or semi-articulated skeletons with other indigestible foods, but the skeleton of a prey animal that had just been swallowed would remain articulated.

**Figure 2:**
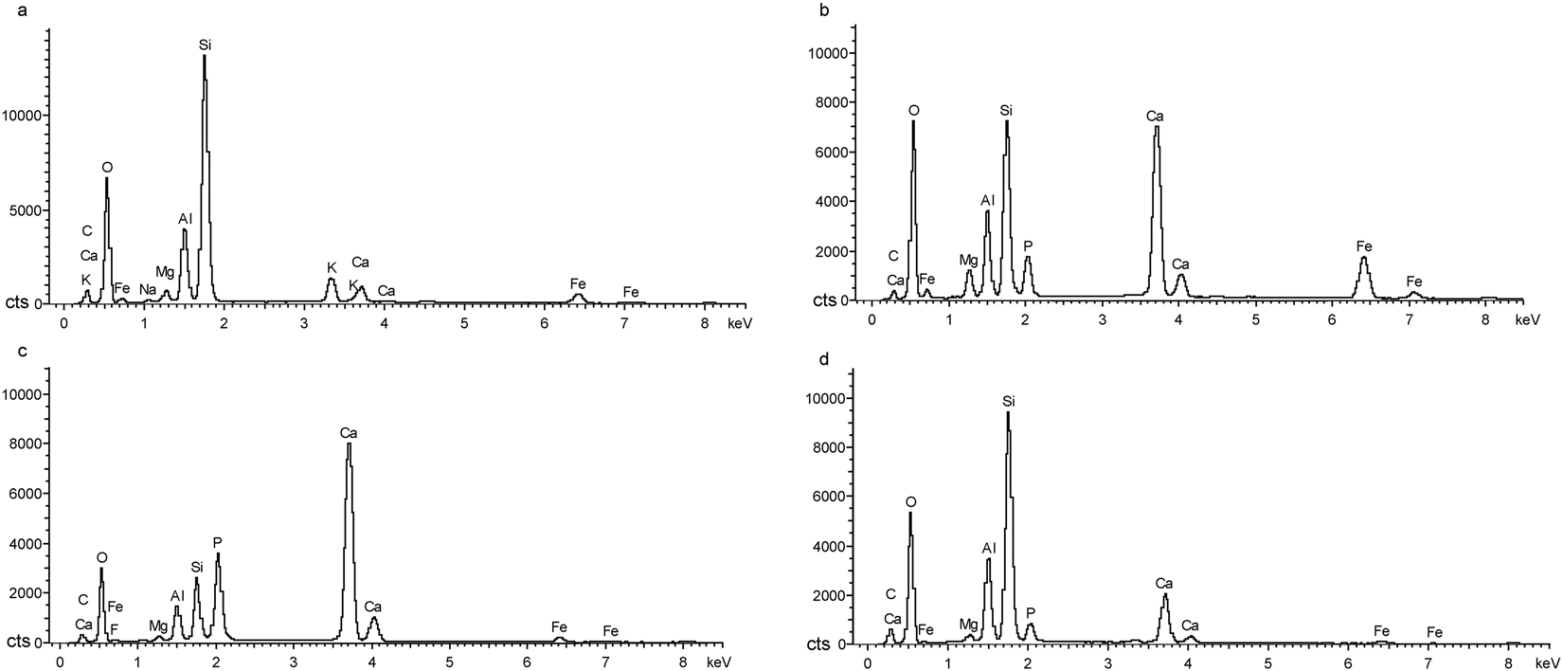
EDS results for the troodontid *Anchiornis* STM0-179. EDS spectra derived from the matrix surrounding the pellet **(a)**, the pellet cement **(b,c**), and a lizard bone contained in the pellet **(d)**.

Finally, we used energy dispersive spectrometry (EDS), a well-established technique for analysing the elemental composition of materials, on four samples from STM0-179. The primary purpose of this analysis was to test the hypothesis that the substance between the lizard bones in the pellet was at least partly derived from dissolution of the bones by stomach acid. If the hypothesis is correct, the interstitial material should display similarities in elemental composition to the lizard bones. The spectra obtained for the two cement samples and the lizard bone sample are closely similar, and differ from the spectrum obtained for the sedimentary matrix surrounding the pellet (Fig. 2). In particular, the spectra for the two cement samples and the lizard bone sample all have large peaks corresponding to calcium and phosphorus, key elemental constituents of the bone-forming mineral hydroxyapatite [3(Ca_3_PO_4_)_2_·Ca(OH)_2_]. These results provide strong confirmation that the material between the lizard bones in the pellet is indeed partly a product of bone dissolution, suggesting that the material represents cement derived from gastric secretions and digestive residues.

**Figure 3:**
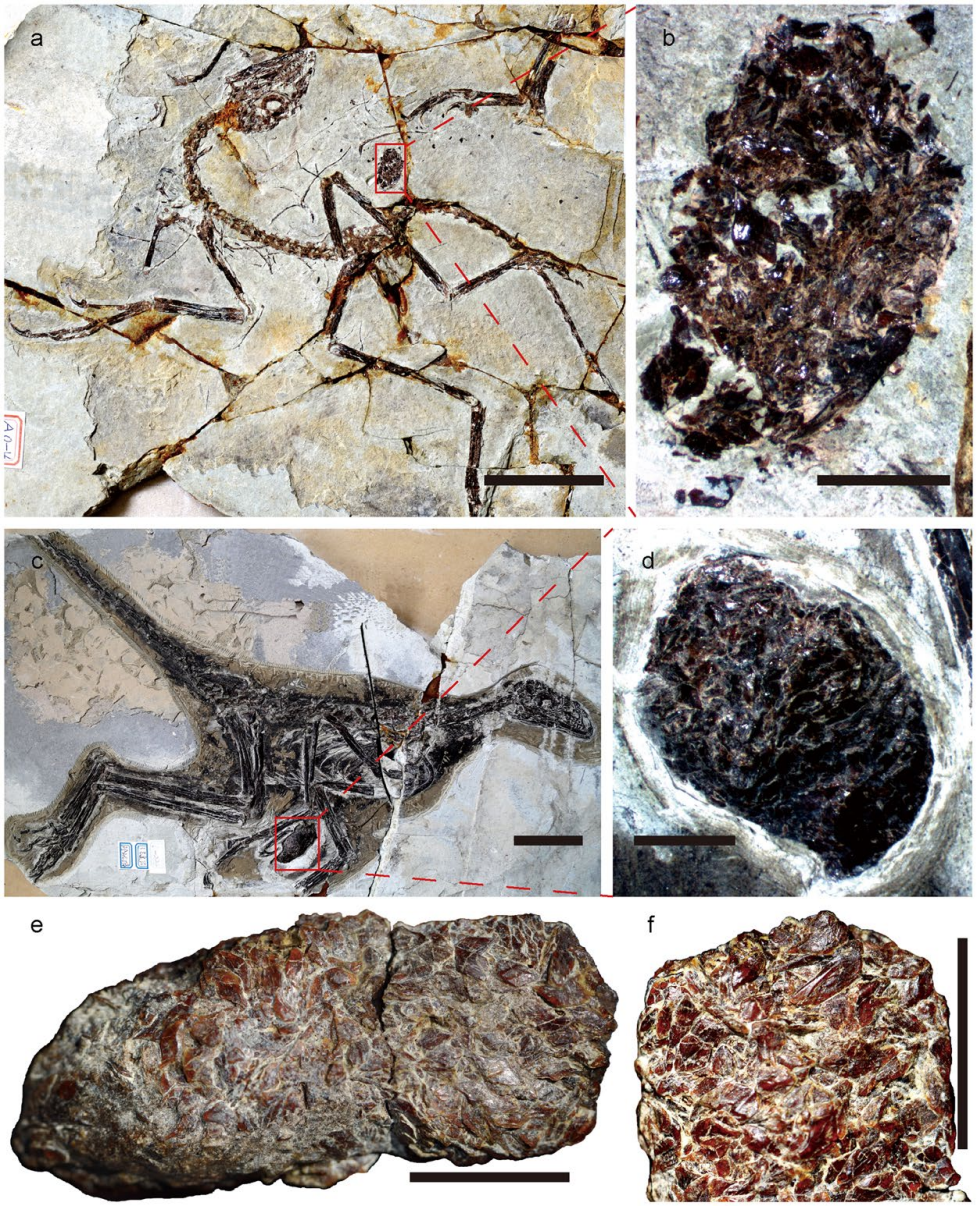
Gastric pellets produced by the troodontid *Anchiornis*, containing fish scales and bones. (**a**) Photograph of *Anchiornis* STMA0-4, with red rectangle framing pellet. (**b)** Close-up of pellet preserved in STMA0-4. (**c)** Photograph of *Anchiornis* STM0-224, with red rectangle framing pellet. **(d)** Close-up of pellet preserved in STM0-224. (**e**) Photograph of isolated pellet STM0-227. **(f**) Close-up of isolated pellet STM0-227. Scale bar, 50 mm for (**a**,**c**), 5 mm for (**b**,**d**–**f**).

In each of the *Anchiornis* specimens STM0-38, STM0-224 and STMA0-4, a highly compact oval structure is preserved near the skeleton (Fig. 3). STM0-227 and STM0-228 are similar structures that were discovered in isolation within the *Anchiornis*-bearing beds. We identify all these objects as pellets, although they differ from the pellet of STM0-179 in being almost entirely composed of ptycholepid fish scales with a few fish bone fragments and little intervening cement. This unusual composition is consistent with the pellet interpretation, but argues against identification of these structures as coprolites. To our knowledge, coprolites likely attributable to predatory archosauromorphs rarely if ever contain such high concentrations of bones and/or scales, and instead are normally rich in cement and other non-skeletal material^7,21–23^. In fact, the objects we identify as *Anchiornis* pellets are fairly similar to probable phorusrhacid pellets from the Miocene of Argentina in being highly calcareous structures with a subovoid shape and a high concentration of indigestible remains, although the phorusrhacid pellets contain fragmentary bones rather than scales^24^. The *Anchiornis* pellets vary in size, the maximum length of STM0-227 (45 mm) being about 2.6 times that of STMA0-4 (17 mm), and our measurements indicate that the sizes of the pellets are positively correlated with those of the associated skeletons (Table 1). The pellet of STMA0-4 appears less compact than the others, and some scales are even scattered across the slab in which this specimen is preserved. The poor consolidation of the pellet may be attributable to weaker gizzard musculature in this ontogenetically less advanced individual, which is about half the size of STM0-179 and -224 and displays poorly fused skeletal elements.

**Table 1:** Selected measurements (in mm) of the *Anchiornis* specimens and their pellets.

Specimen	Femur length	Pellet long axis	Pellet short axis
STM0-224	70	39	18
STM0-179	71	53	20
STMA0-4	35*	17	11
STM0-38	40.5	30	13
STM0-227		45	20
STM0-228		35	20

## Discussion

The discovery of gastric pellets in the basal troodontid *Anchiornis* sheds new light on the evolution of the avian digestive system. Previous studies have demonstrated that non-avialan theropods share with modern birds some salient features pertaining to feeding and digestion. Like carnivorous birds, carnivorous non-avialan theropods probably swallowed prey whole or in large chunks^9^, as indicated by stomach contents in several theropod fossils that include partial or even nearly complete skeletons of a variety of small vertebrates^9,17,25–27^. The presence of gastroliths in several phylogenetically disparate theropod groups, including ceratosaurs^28^, ornithomimosaurs^29,30^, and oviraptorosaurs^31,32^, suggests that theropods probably had a two-chambered stomach with a muscular gizzard^3,10^. However, relatively basal coelurosaurian theropods probably differed from most birds in several digestive features. For example, the compsognathids *Sinocalliopteryx*^17^ and *Scipionyx*^18^ might have possessed highly acidic anterior stomach chambers, as indicated by the occurrence in these taxa of strongly acid-etched bones as apparent preserved stomach contents^18^. Furthermore, the strong etching also suggests that these basal coelurosaurian theropods may have been characterized by relatively long gastric residence times similar to those of modern crocodilians^33^.

The *Anchiornis* gastric pellets described in this study are the only ones definitively known from any non-avialan theropod, though an isolated aggregation of bones from the Lower Cretaceous of Las Hoyas, Spain has been identified as a pellet from either a non-avialan theropod or a pterosaur^19^. If the lack of other documented fossil theropod pellets is not simply a preservational artefact, parsimony suggests that pellet regurgitation and the advanced digestive features (a two-chambered stomach, efficient anti-peristalsis, low stomach acidity and short gastric residence) implied by this phenomenon were absent in non-paravians and evolved at the base of Paraves. However, gastric pellets of any kind have rarely been reported in the fossil record^20^, particularly in circumstances that permit them to be assigned to particular taxa, so the possibility that pellets occurred in non-paravians but have not yet been successfully recovered and identified must be considered.

Although the evidence bearing on whether pellet regurgitation might have occurred in some non-paravians is scattered and largely circumstantial, some tentative conclusions can be drawn. The most important datum is the occurrence of bone fragments in coprolites attributable to the basal (non-maniraptoriform) coelurosaur *Tyrannosaurus*^7–9^, which implies that hard indigestible material was still being expelled via the cloaca rather than orally in taxa of this evolutionary grade. There is no known evidence of either gastric pellets or bone-bearing coprolites in the basal coelurosaurian clade Compsognathidae, or in the non-paravian maniraptoriform clades Ornithomimosauria, Alvarezsauroidea, Therizinosauroidea and Oviraptorosauria. However, an important consideration in evaluating whether any of these taxa are likely to have produced gastric pellets is their inferred diet. Compsognathids were clearly carnivorous, based on their dentition and the occurrence of vertebrate remains as stomach contents in some compsognathid specimens^17,34–36^. However, ornithomimosaurs, therizinosauroids and oviraptorosaurs are all characterized by reduced or lanceolate teeth and other craniomandibular indicators of herbivory, and some ornithomimosaurs and oviraptorosaurs also have gastric mills that likely represent an additional adaptation for plant-eating^37^. Although the teeth of alvarezsauroids are reduced in size, some maxillary teeth of the basal form *Haplocheirus* are recurved and serrated as in undoubtedly faunivorous theropods^38,39^, and insectivory has been suggested for derived members of the group^40,41^. Alvarezsauroids appear much less likely than other non-paravian maniraptorans to have been largely or entirely herbivorous, but even *Haplocheirus* lacked skull and dental features suggestive of classic theropod carnivory and was presumably restricted to prey much smaller than itself^39^.

Among extant birds, pellet regurgitation is characteristic of carnivores, piscivores and insectivores^20^. Herbivores presumably either digest their food completely, or expel indigestible material in the faeces. If this pattern was also characteristic of non-avian theropods, then ornithomimosaurs, therizinosaurs and oviraptorosaurs probably did not produce gastric pellets, given their inferred herbivory. However, the presence of gastric mills in some ornithomimosaurs and oviraptorosaurs suggests that a muscular, grinding gizzard was present ancestrally in maniraptoriforms^3^. Although a muscular gizzard is necessary in order to form gastric pellets, the presence of a gizzard does not necessarily imply that a given theropod was capable of the efficient antiperistalsis needed for pellet regurgitation. Because of the evidence for cloacal rather than oral expulsion of indigestible residue in tyrannosauroids^7^, efficient antiperistalsis was probably absent in non-maniraptoriform theropods, and this feature of the digestive system was probably also plesiomorphically absent in the herbivorous Ornithomimosauria. Alvarezsauroids, which are probably less closely related to birds than are therizinosauroids and oviraptorosaurs^39^, are the most basal faunivorous maniraptoriforms. Because no alvarezsauroid coprolites or pellets have ever been reported, it is uncertain whether they digested their prey completely, expelled indigestible material cloacally, or expelled indigestible material orally.

Combining the new evidence from *Anchiornis* with previous information on theropod digestion suggests two plausible scenarios for the evolution of efficient antiperistalsis and the tendency to produce gastric pellets. One possibility is that efficient antiperistalsis is a primitive maniraptoran feature, which would imply that oral regurgitation occurred in at least some alvarezsauroids. Therizinosaurs and oviraptorosaurs would then have been capable of this type of antiperistalsis, at least plesiomorphically, but would probably have needed to resort to it only in unusual circumstances given their herbivorous diets. *Anchiornis* and at least some other faunivorous basal paravians, by contrast, would have used their inherited capacity for efficient antiperistalsis to rid themselves of bones and other indigestible prey residues through oral regurgitation. The alternative evolutionary possibility is that alvarezsauroids still lacked efficient antiperistalsis, which really did emerge at the origin of Paraves as suggested by the absence of known pellets from more basal theropods. It will be likely be impossible to judge between the two scenarios until alvarezsauroid coprolites and/or pellets are available for analysis.

**Figure 4:**
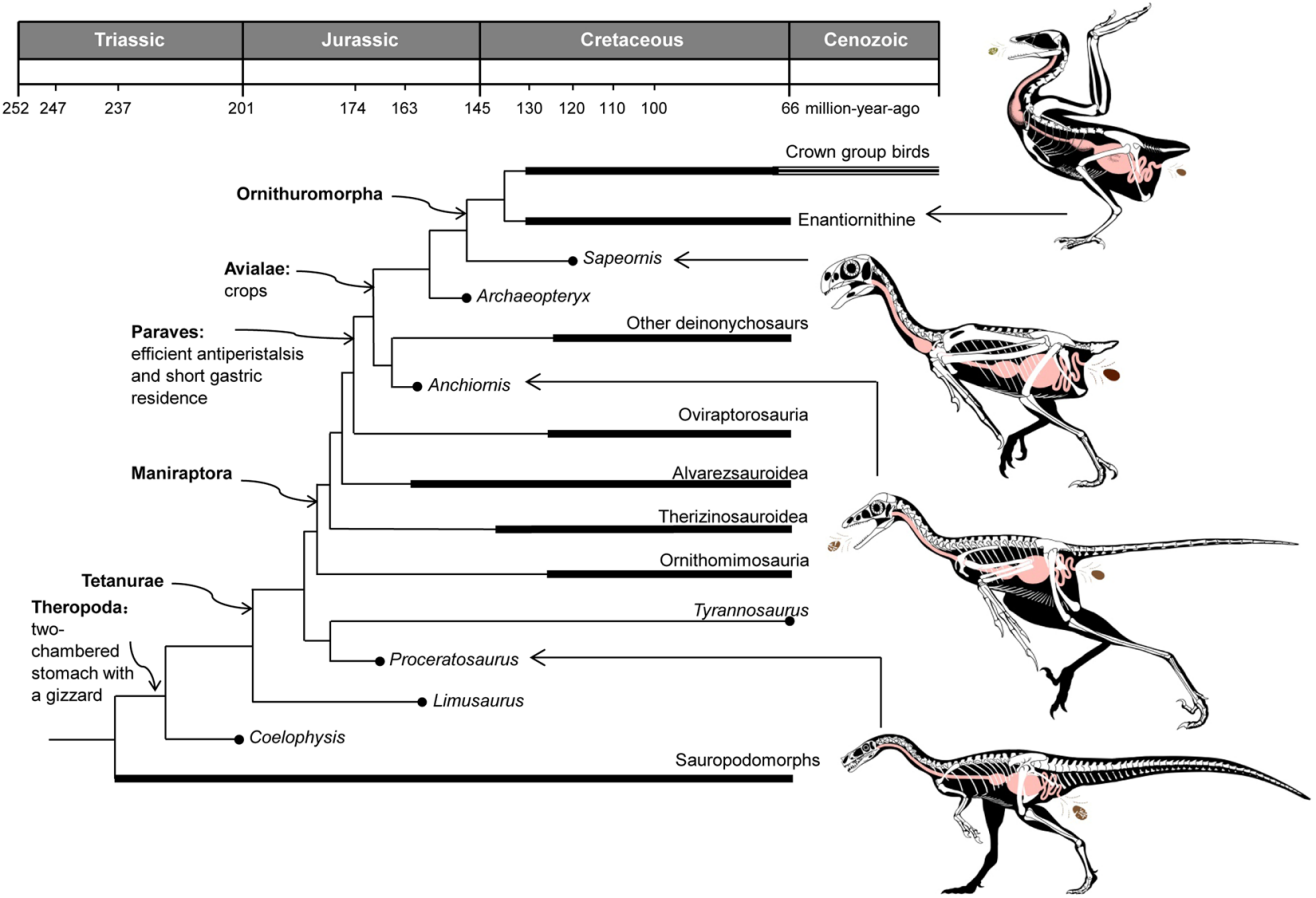
Evolution of digestive features in theropod dinosaurs. A two-chambered stomach with muscular gizzard, possibly highly acidic anterior stomach chamber, and relatively long gastric residence time might have characterized most theropods, including basal coelurosaurians; a digestive system with relatively short residence time and efficient antiperistalsis to expel indigestible material orally might have originated at the base of Paraves; and some highly specialized digestive structures such as the crop might have originated at the base of the Pygostylia.

The *Anchiornis* pellets described here are highly similar to those of modern birds. The bones and/or scales in all these pellets retain relatively smooth surfaces and show no signs of strong acid-etching, suggesting short gastric residence as in most modern birds^20^. In both temporal and phylogenetic terms, the basal troodontid *Anchiornis* represents the earliest theropod that can be shown to have possessed both a two-chambered stomach, efficient antiperistalsis, low stomach acidity and short gastric residence, suggesting that the highly efficient and specialized avian digestive system was plesiomorphically present in the Paraves or even the Maniraptora (Fig. 4). In particular, oral expulsion would have greatly improved digestive efficiency^20^, which could have helped provide the energy needed for aerial locomotion, and early paravians could perhaps also have slightly reduced their body mass by quickly expelling any ingested material that was resistant to digestion. A number of other features critical for aerial locomotion are also thought to have appeared at the base of the Paraves, including cerebral expansion and elaboration of visually associated brain regions^42^, forelimb enlargement^16,43^, and a more metabolically active physiology^44^. If such an efficient digestive system indeed originated at the base of the Paraves in connection with the above-mentioned biological innovations, it will provide further support for the appearance of aerial locomotion in basal paravians^16,44,45^.

The presence of both terrestrial lizards and aquatic fish in the diet of *Anchiornis* suggests that this dinosaur was an opportunistic generalist hunter. *Anchiornis* would then have been broadly similar in its foraging behaviour to the dromaeosaurid *Microraptor*, which evidently fed on mammals, birds, and fish^25–27^. The presence of three lizard skeletons in the pellet of STM0-179 indicates that *Anchiornis* must have consumed three prey animals in rapid succession. Preserved stomach contents of a referred specimen of the compsognathid *Sinocalliopteryx* also include multiple prey items^17^.

However, the pellet contents of *Anchiornis* differ from the stomach contents of *Microraptor* and *Sinocalliopteryx* in including a particularly high proportion of fish, with five pellets of the six described here containing only fish scales. The preponderance of fish scales in gastric pellets attributable to *Anchiornis*, if taken at face value, suggests that fish formed an important or even predominant component of this dinosaur’s diet. This observation is surprising given that *Anchiornis* does not, based on comparisons to its living relatives, appear well suited to catching fish or even living in close proximity to water. *Anchiornis* has extensive feathering on the lower legs, including the toes, whereas extant near-shore or aquatic birds tend to have little plumage below the knee. Furthermore, *Anchiornis* has a relatively short snout, whereas fish-catching birds usually have long, slender bills.

Taken together, evidence from the newly reported pellets and from the morphology of *Anchiornis*, particularly when the latter is considered in the light of comparative data from extant birds, creates the seemingly paradoxical picture of an animal that had a mainly piscivorous diet but was nevertheless poorly adapted to catching fish. We suggest that this paradox may have a behavioural and/or taphonomic resolution. Behaviourally, *Anchiornis* may simply have been good *enough* at obtaining fish to thrive as a partial piscivore, despite appearing poorly suited to fishing. Taphonomically, some kind of bias may have favoured the preservation of fish-bearing pellets over those containing the remains of terrestrial vertebrates, so that the pellet evidence is not really representative of the normal diet of *Anchiornis*. Nevertheless, what can be stated definitively is that the diet of *Anchiornis* included both fish and lizards at least on an occasional basis, and that *Anchiornis* possessed a derived digestive system capable of forming and regurgitating pellets in an essentially avian manner.

## Methods

### Morphological study

The fossils were prepared by mechanical methods using pneumatic preparation tools. Morphological observations were made using both the naked eye and a microscope (Zeiss Stemi 2000-c). Photographs were taken with an ordinary camera (Nikon D800) as well as one mounted on a digital microscope (Zeiss SteREO V20). Although a recent study has provided a classification of vertebrate coprolites and other similar trace fossils^46^, the proposed terminology has not been widely accepted. Here we adopt more widely used terms for digestive traces: gastric pellets (masses of indigestible food material compacted in the muscular gizzard and expelled from the mouth; characterized by an extremely high percentage of indigestible material such as bones and feathers and a low percentage of cement derived from stomach residues and broken down food material), coprolites (mostly digestible food material expelled from the cloaca or anus; characterized by a low percentage of indigestible material such as bones and feathers and a high percentage of cement derived from gastrointestinal residues and highly broken down food material), and gut contents (food material remaining inside the stomach or intestines; more similar in composition to coprolites than to pellets).

### Energy dispersive spectrometry (EDS) analysis

In EDS, a beam of X-rays or charged particles is used to cause a sample to fluoresce, and the resulting radiation is analysed in terms of the energy distribution of the photons produced. Peaks on a spectrum of photon energies correspond to particular elements present in the sample, as different elements characteristically emit photons of different energy levels. Four samples were taken from STM0-179, including one from a piece of lizard bone, one from the sedimentary matrix surrounding the pellet, and two from the supposed pellet cement. None of the samples was coated prior to analysis. The samples were analysed using a Hitachi S-3400N scanning electron microscope (SEM) at Linyi University, with the following parameters: accelerating voltage 25 keV, working distance 10.4 mm and live time 40 seconds.

## References

[1] Duke GE (1997). Gastrointestinal physiology and nutrition in wild birds. Proceedings of the Nutrition Society.

[2] McLelland, J. In *Form and Function in* Birds (eds King, A. S. & McLelland, J.) 69–181 (Academic Press, 1979).

[3] Wang Min, Zhou Zhonghe, Sullivan Corwin (2016). A Fish-Eating Enantiornithine Bird from the Early Cretaceous of China Provides Evidence of Modern Avian Digestive Features. Current Biology.

[4] Zheng X (2011). Fossil evidence of avian crops from the Early Cretaceous of China. Proceedings of National Academy of Sciences USA.

[5] O’Connor JK, Zhou ZH (2015). Early evolution of the biological bird: perspectives from new fossil discoveries in China. Journal of Ornithology.

[6] Naish D (2014). The fossil record of bird behaviour. Journal of Zoology.

[7] Chin K, Tokaryk TT, Erickson GM, Calk LC (1998). A king-sized theropod coprolite. Nature.

[8] Hone DWE, Rauhut OWM (2010). Feeding behaviour and bone utilization by theropod dinosaurs. Lethaia.

[9] Varricchio DJ (2001). Gut contents from a Cretaceous tyrannosaurid; implications for theropod dinosaur digestive tracts. Journal of Paleontology.

[10] Wings, O. *Identification, distribution, and function of gastroliths in dinosaurs and extant birds with emphasis on ostriches (Struthio camelus)*, The University of Bonn (2004).

[11] Liu YQ (2012). Timing of the earliest known feathered dinosaurs and transitional pterosaurs older than the Jehol Biota. Palaeogeography Palaeoclimatology Palaeoecology.

[12] Xu X (2009). A new feathered maniraptoran dinosaur fossil that fills a morphological gap in avian origin. Chinese Science Bulletin.

[13] Hu DY, Hou L-H, Zhang LJ, Xu X (2009). A pre-*Archaeopteryx* troodontid from China with long feathers on the metatarsus. Nature.

[14] Li QG (2010). Plumage color patterns of an extinct dinosaur. Science.

[15] Brusatte S, Lloyd G, Wang S, Norell M (2014). Gradual assembly of avian body plan culminated in rapid rates of evolution across the dinosaur-bird transition. Current Biology.

[16] Xu X, You H, Du K, Han F (2011). An *Archaeopteryx*-like theropod from China and the origin of Avialae. Nature.

[17] Xing L (2012). Abdominal contents from two large Early Cretaceous compsognathids (Dinosauria: Theropoda) demonstrate feeding on confuciusornithids and dromaeosaurids. PLoS ONE.

[18] Sasso CD, Maganuco S (2011). *Scipionyx samniticus* (Theropoda: Compsognathidae) from the Lower Cretaceous of Italy — Osteology, ontogenetic assessment, phylogeny, soft tissue anatomy, taphonomy and palaeobiology. Memorie della Società Italiana de Scienze Naturali e del Museo Civico di Storia Naturale di Milano.

[19] Sanz JL (2001). A Early Cretaceous pellet. Nature.

[20] Myhrvold NP (2013). A call to search for fossilised gastric pellets. Historical Biology.

[21] Hollocher KT, Alcober OA, Colombi CE, Hollocher TC (2005). Carnivore coprolites from the Upper Triassic Ischigualasto Formation, Argentina: chemistry, mineralogy, and evidence for rapid initial mineralization. Palaios.

[22] Northwood C (2005). Early Triassic coprolites from Australia and their palaeobiological significance. Palaeontology.

[23] Zatoń M (2015). Coprolites of Late Triassic carnivorous vertebrates from Poland: An integrative approach. Palaeogeography, Palaeoclimatology, Palaeoecology.

[24] Nasif NL, Esteban GI, Ortiz PE (2009). Novedoso hallazgo de egagrópilas en el Mioceno tardío, Formación Andalhuala,provincia de Catamarca, Argentina. Serie Correlación Geológica.

[25] O’Connor J, Zhou ZH, Xu X (2011). Additional specimen of *Microraptor* provides unique evidence of dinosaurs preying on birds. Proceedings of the National Academy of Science, USA.

[26] Xing L-D (2013). Piscivory in the feathered dinosaur *Microraptor*. Evolution.

[27] Larsson H, Hone D, Dececchi T, Sullivan C, Xu X (2010). The winged non-avian dinosaur Microraptor fed on mammals: Implications for the Jehol Biota ecosystem. Journal of Vertebrate Paleontology.

[28] Xu X (2009). A Jurassic ceratosaur from China helps clarify avian digit homologies. Nature.

[29] Makovicky, P. J., Kobayashi, Y. & Currie, P. J. In *The Dinosauria (second edition)* (eds Weishampel, D. B., Dodson, P. & Osmolska, H.) 137–150 (University of California Press, 2004).

[30] Kobayashi Y (1999). Herbivorous diet in an ornithomimid dinosaur. Nature.

[31] Osmólska, H., Currie, P. J. & Barsbold, R. In *The Dinosauria* 2nd *edn* (eds Weishampel, D. B., Dodson, P. & Osmólska, H.) 165–183 (University of California Press, 2004).

[32] Zhou Z-H, Wang X-L, Zhang F-C, Xu X (2000). Important features of *Caudipteryx*-evidence from two nearly complete new specimens. Vertebrata PalAsiatica.

[33] Farmer C, Uriona T, Olsen D, Steenblik M, Sanders K (2008). The right-to left shunt of crocodilians serves digestion. Physilogical and Biochemical Zoology.

[34] Ostrom JH (1978). The osteology of *Compsognathus logipes* Wagner. Zitteliana.

[35] Chen PJ, Dong ZM, Zhen SN (1998). An exceptionally well-preserved Theropod dinosaur from the Yixian Formation of China. Nature.

[36] Ji SA, Ji Q, Lu JC, Yuan CX (2007). A new gigant compsognathid dinosaur with long filamentous integuments from Lower Cretaceous of Northeastern China. Acta Geologica Sinica.

[37] Zanno LE, Makovicky PJ (2011). Herbivorous ecomorphology and specialization patterns in theropod dinosaur evolution. Proceedings of the National Academy of Sciences of the United States of America.

[38] Choiniere JN (2010). A basal alvarezsauroid theropod from the early Late Jurassic of Xinjiang, China. Science.

[39] Choiniere J, Clark JM, Norell MA, Xu X (2014). Cranial osteology of *Haplocheirus sollers* Choiniere *et al*., 2010 (Theropoda: Alvarezsauroidea). American Museum Novitates.

[40] Senter P (2005). Function in the stunted forelimbs of *Mononykus olecranus* (Theropoda), a dinosaurian anteater. Paleobiology.

[41] Xu X (2010). A basal parvicursorine (Theropoda: Alvarezsauridae) from the Upper Cretaceous of China. Zootaxa.

[42] Balanoff A, Bever G, Rowe T, Norell MA (2013). Evolutionary origins of the avian brain. Nature.

[43] Allen V, Bates KT, Li ZH, Hutchinson JR (2013). Linking the evolution of body shape and locomotor biomechanics in bird-line archosaurs. Nature.

[44] Li Q (2014). Melanosome evolution indicates a key physiological shift within feathered dinosaurs. Nature.

[45] Xu X (2014). An integrative approach to understanding bird origins. Science.

[46] Hunt AP, Lucas SG (2012). Classification of vertebrate coprolites and related trace fossils. New Mexico Museum of Natural History and Science Bulletin.

